# Albumin Levels and the Risk of Heart Failure in Patients Undergoing Coronary Angiography for Different Indications

**DOI:** 10.3390/jcm15176594

**Published:** 2026-08-26

**Authors:** Henning Johann Steffen, Michael Behnes, Lasse Kuhn, Philipp Steinke, Mahboubeh Jannesari, Fabian Siegel, Ibrahim Akin, Tobias Schupp

**Affiliations:** 1 Cardiology, Hemostasis, and Medical Intensive Care, University Medical Center Mannheim, Medical Faculty Mannheim, Heidelberg University, Theodor-Kutzer-Ufer 1-3, 68167 Mannheim, Germany; henningjohann.steffen@umm.de (H.J.S.);; 2 Department of Biomedical Informatics, Center for Preventive Medicine and Digital Health (CPD), Medical Faculty Mannheim, Heidelberg University, Theodor-Kutzer-Ufer 1-3, 68167 Mannheim, Germany

**Keywords:** albumin, hypoalbuminemia, coronary angiography, heart failure, prognosis

## Abstract

**Background/Objective:** Albumin has emerged as a prognostic marker across cardiovascular disease, but its independent association with heart failure (HF) in different clinical settings remains scarce. This study investigated the prognostic impact of baseline albumin levels in patients undergoing coronary angiography (CA) for different indications. **Methods:** Consecutive inpatients undergoing CA from 2016 to 2022 were retrospectively included at one institution and stratified into quartiles according to baseline albumin levels (Q1: <30, Q2: 30–34, Q3: 34–37, Q4: >37 g/L). The primary endpoint was rehospitalization for HF at 36 months; secondary endpoints were coronary revascularization and acute myocardial infarction (AMI). Statistical analyses included Kaplan–Meier and Cox regression analyses. **Results:** A total of 7520 patients were included (median albumin: 34.3 g/L; IQR: 30.5–37.2 g/L). Lower albumin levels were associated with a higher comorbidity burden and more extensive coronary artery disease (three-vessel disease: Q1: 36.1% vs. Q4: 21.4%; *p* = 0.001). HF-related rehospitalization at 36 months increased progressively with lower albumin (Q1: 28.4% vs. Q2: 25.1% vs. Q3: 21.0% vs. Q4: 13.9%; *p* = 0.001). After multivariable adjustments, the lowest albumin group (<30 g/L) remained independently associated with HF-related rehospitalization (HR = 1.381; 95% CI: 1.105–1.727; *p* = 0.005), whereas no independent association with coronary revascularization or AMI was demonstrated. The association was most evident in patients ≥70 years of age and in those with left ventricular ejection fraction (LVEF) ≥ 35%. **Conclusions:** In unselected patients undergoing CA, lower albumin levels independently predicted the HF-related rehospitalization at 36 months, but not coronary revascularization or AMI. Routine albumin measurement may provide meaningful prognostic information in patients undergoing CA.

## 1. Introduction

Serum albumin, the most abundant circulating plasma protein, has emerged as a prognostic marker across a broad spectrum of cardiovascular disease due to its pleiotropic roles in maintaining endothelial stability, modulating inflammatory responses, and buffering oxidative stress [1,2,3]. Importantly, this relationship appears unlikely to be purely epiphenomenal: a recent Mendelian randomization study found that genetically predicted lower serum albumin was causally associated with increased cardiovascular disease risk, supporting the role of albumin as a causally relevant marker rather than merely a downstream reflection of illness severity [4]. Yet its independent predictive value in unselected patients undergoing coronary angiography (CA) remains incompletely defined.

Mounting evidence implicates hypoalbuminemia as a predictor of adverse cardiovascular outcomes across diverse settings. In acute coronary syndrome (ACS), lower albumin levels at presentation are associated with higher in-hospital and long-term mortality [5,6]. In patients with stable coronary artery disease (CAD) undergoing percutaneous coronary intervention (PCI), albumin independently predicts long-term adverse outcomes including cardiovascular death and heart failure (HF) hospitalization [7,8]. Moreover, in HF populations, albumin levels strongly correlate with prognosis, reflecting both cardiac cachexia and systemic inflammation [9,10], and more recently has been shown to independently predict mortality in HF and cardiogenic shock [11,12].

Despite these observations, serum albumin has not been integrated into the current cardiovascular risk algorithms. This may partly reflect the methodological limitations of previous studies, which often enrolled selected populations, such as those with advanced HF [13], or chronic kidney disease (CKD) [14], and failed to adjust for modern angiographic and procedural variables. Furthermore, few large-scale studies have assessed albumin’s independent prognostic value in unselected patients undergoing CA, where outcomes such as HF-related rehospitalization, acute myocardial infarction (AMI), and coronary revascularization at 36 months affect a substantial proportion of patients and carry important prognostic implications.

The present study aims to evaluate the independent prognostic association of baseline albumin levels with regard to the risks of HF-related rehospitalization, coronary revascularization, and AMI at 36 months in a large, consecutive, unselected cohort of inpatients undergoing CA, with pre-specified subgroup analyses to identify the clinical contexts in which albumin most strongly predicts outcome. Given that albumin predominantly reflects inflammatory, nutritional, and neurohormonal pathways rather than plaque instability or thrombotic risk, we hypothesized that albumin would preferentially predict HF-related rather than ischemic endpoints in this setting.

## 2. Materials and Methods

### 2.1. Study Patients, Design, and Data Collection

For the present study, all consecutive inpatients undergoing CA at the University Medical Center Mannheim (UMM), Germany were included from January 2016 to August 2022. Patients were identified using the German Operation and Procedure Classification (OPS) codes. The local electronic hospital information system (SAP^®^, Walldorf, Germany) was used to retrospectively extract all relevant clinical data related to the index hospitalization, including admission symptoms, primary diagnoses, medical history, angiographic findings, performed interventions, and discharge medications. Patients who underwent recurrent CA were only included once. The UMM provides a 24/7 cardiac catheterization laboratory, an electrophysiological laboratory, a hybrid operating suite, and telemetry units. The present study was derived from a retrospective single-center registry including consecutive patients undergoing CA, hospitalized at the University Medical Center Mannheim (UMM), Germany (DRKS-ID: DRKS00034765). The registry was established in accordance with the Declaration of Helsinki and approved by the Medical Ethics Committee II of the Medical Faculty Mannheim, University of Heidelberg, Germany (ethics approval code: 2022-829; date of approval: 19 May 2022).

### 2.2. Inclusion and Exclusion Criteria

For the present study, all consecutive patients aged ≥ 18 years who underwent invasive CA at the UMM, Germany between January 2016 and August 2022 were eligible for inclusion. CA operators were blinded to the final study analyses. All source data of the CA examinations (imaging files) and reports were reassessed post hoc by two independent cardiologists. Patients younger than 18 years and those without an albumin measurement during index hospitalization were excluded. Patients who died during the index hospitalization were not included in the outcome analysis. Risk stratification was performed according to the baseline albumin levels. Patients were stratified into four groups according to quartiles.

### 2.3. Measurement of Albumin Levels

After adequate clotting of serum samples for 1 h at room temperature, samples were centrifuged at 2000× *g* for 10 min at 18 °C according to the manufacturer’s recommendations. Albumin measurements were performed fully automatically by extinction measurement based on the color binding method according to Carter and Louderback (Atellica CH 930; Siemens Healthineers, Erlangen, Germany). The reference range for albumin in adults was 34–50 g/L. The detection limit (LoD) of the method was 6 g/L, and the assay showed a linearity range from 5–80 g/L. All investigations were carried out at an accredited laboratory under DIN EN ISO 15189 conditions [12]. For the present study, the most recent albumin levels obtained prior to CA during the index hospitalization was used for all analyses; all measurements were performed on venous samples collected as part of routine clinical workup. In patients with multiple albumin measurements, the value closest to the date of index CA was selected. All albumin values were obtained during the same hospital stay as the index CA, with the most proximal available measurement prior to CA selected for analysis; the interval between sampling and CA was therefore consistently short, generally within a few days.

### 2.4. Study Endpoints

The primary endpoint was rehospitalization for HF within 36 months following the index CA. Secondary endpoints included coronary revascularization and AMI within 36 months. All endpoints were defined using International Classification of Diseases (ICD) codes at the UMM, based on hospital records without independent, cross-institutional endpoint adjudication.

### 2.5. Statistical Methods

Statistical analyses were performed using SPSS Statistics (Version 25, IBM Corp., Armonk, NY, USA). Continuous variables were presented as medians and interquartile ranges (IQR), whereas categorical variables are presented as frequencies and percentages. Continuous variables were compared using the Kruskal–Wallis test and categorical variables using the Chi-square test. The primary endpoint of HF-related rehospitalization at 36 months and the secondary endpoints of coronary revascularization and AMI at 36 months were analyzed according to the baseline albumin groups. Event-free survival was estimated using Kaplan–Meier analyses and compared by the log-rank test. Univariable and multivariable Cox proportional hazards regression models were performed to identify predictors of HF-related rehospitalization, coronary revascularization, and AMI at 36 months. Multivariable models included the albumin quartile as the primary exposure variable together with pre-specified covariates. Hazard ratios (HRs) and 95% confidence intervals (CIs) were calculated. Multivariable models were adjusted for age, sex, body mass index (BMI), diabetes mellitus, CKD (estimated glomerular filtration rate (eGFR) < 60 mL/min/1.73 m^2^), anemia, C-reactive protein (CRP) levels, malignancies, atrial fibrillation (AF), ST-segment elevation myocardial infarction (STEMI), non-ST-segment elevation myocardial infarction (NSTEMI), acute decompensated HF (ADHF), and left ventricular ejection fraction (LVEF) < 35%. Anemia was defined according to the World Health Organization (WHO) criteria as a hemoglobin level < 13 g/dL in men and <12 g/dL in women. Furthermore, predefined subgroup analyses were performed using multivariable Cox regression models to evaluate the association of albumin levels and HF-related rehospitalization at 36 months. In a sensitivity analysis, albumin was alternatively modeled as a continuous, log-transformed (ln-albumin) variable. Predefined subgroup analyses were performed by age (<70 vs. ≥70 years), sex, CAD extent (0- to 1-vessel vs. multivessel disease), LVEF (<35% vs. ≥35%), indication acuity (elective vs. acute, the latter defined as unstable angina, NSTEMI, STEMI, resuscitated cardiac arrest, or cardiogenic shock), STEMI status, and ADHF status. For each subgroup, a formal interaction term (ln-albumin x subgroup) was calculated using logistic regression models for HF-related rehospitalization, and a Bonferroni-corrected significance threshold (α = 0.05/7 = 0.0071) was applied to account for the seven comparisons performed. Discrimination for HF-related rehospitalization was assessed by receiver operating characteristic (ROC) curve analysis, comparing the area under the curve (AUC) for albumin alone, the AHEAD risk score (Atrial fibrillation, Hemoglobin, Elderly age, Abnormal renal parameters, and Diabetes mellitus [15], and the AHEAD score with albumin added; albumin was dichotomized at its optimal cutoff by the Youden index for this analysis. All statistical tests were two-tailed and a *p*-value ≤ 0.05 was considered statistically significant.

## 3. Results

### 3.1. Study Population

**Figure 1:**
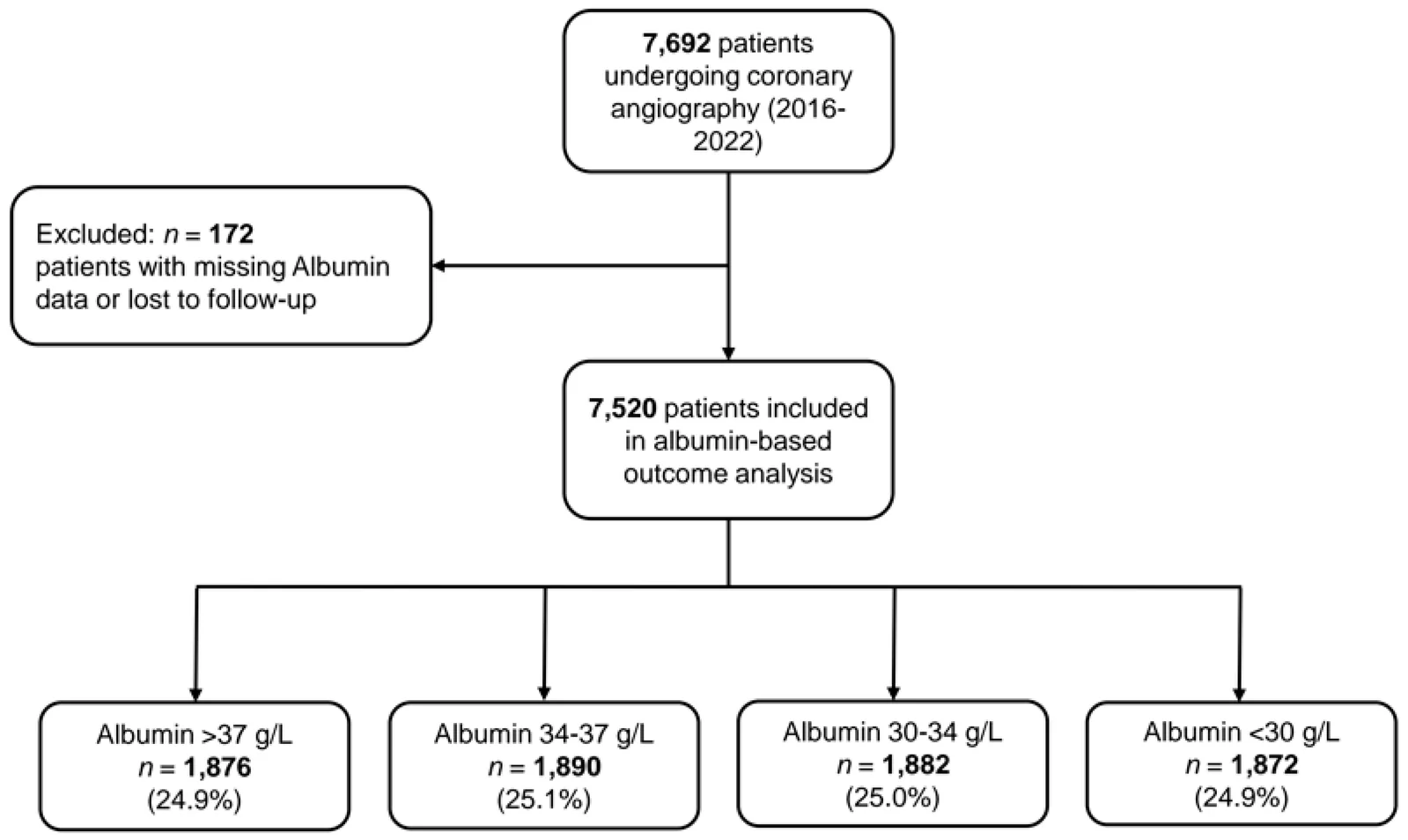
Flowchart illustrating formation of the study cohort including patient exclusion and stratification into four albumin quartiles: Q1 (<30 g/L), Q2 (30–34 g/L), Q3 (34–37 g/L), and Q4 (>37 g/L).

Between January 2016 and August 2022, a total of 7692 inpatients underwent CA at the catheterization unit of the UMM. A total of 172 patients were excluded due to missing albumin data. The final study cohort comprised 7520 patients. Serum albumin levels in the overall cohort had a median of 34.3 g/L (mean: 33.4 g/L; IQR: 30.5–37.2 g/L). Patients were stratified into four quartiles (Q1–Q4) based on the baseline serum albumin levels: Q1 (albumin < 30 g/L; *n* = 1872; 24.9%), Q2 (albumin 30–34 g/L; *n* = 1882; 25.0%), Q3 (albumin 34–37 g/L; *n* = 1890; 25.1%), and Q4 (albumin > 37 g/L; *n* = 1876; 24.9%) (Figure 1). Indications for CA differed significantly across albumin quartiles (Table 1): the prevalence of STEMI decreased from 15.2% in Q1 to 6.7% in Q4, while unstable angina increased from 10.1% in Q1 to 42.3% in Q4 (both *p* = 0.001).

Patients with lower albumin levels were older (median age: Q1: 73 years vs. Q4: 64 years; *p* = 0.001) and less frequently male (Q1: 62.2% vs. Q4: 70.0%; *p* = 0.001). BMI decreased with lower albumin levels (Q1: 26 kg/m^2^ vs. Q4: 28 kg/m^2^; *p* = 0.001). With regard to cardiovascular risk factors, diabetes mellitus (Q1: 35.4% vs. Q4: 23.5%; *p* = 0.001) and arterial hypertension (Q1: 74.6% vs. Q4: 86.2%; *p* = 0.001) differed significantly across groups, while hyperlipidemia was more prevalent with higher albumin levels (Q1: 23.8% vs. Q4: 44.8%; *p* = 0.001). The rates of prior congestive HF (Q1: 11.4% vs. Q4: 6.9%; *p* = 0.001), CKD (Q1: 9.9% vs. Q4: 2.1%; *p* = 0.001), chronic obstructive pulmonary disease (COPD) (Q1: 6.1% vs. Q4: 2.1%; *p* = 0.001), and malignancy (Q1: 9.6% vs. Q4: 3.2%; *p* = 0.001) increased with decreasing albumin levels.

Regarding comorbidities at index hospitalization, STEMI (Q1: 15.2% vs. Q4: 6.7%; *p* = 0.001), NSTEMI (Q1: 23.5% vs. Q4: 11.8%; *p* = 0.001), AF (Q1: 30.8% vs. Q4: 21.9%; *p* = 0.001), ADHF (Q1: 19.5% vs. Q4: 5.0%; *p* = 0.001), cardiogenic shock (Q1: 13.4% vs. Q4: 0.6%; *p* = 0.001), and cardiopulmonary resuscitation (Q1: 22.0% vs. Q4: 0.9%; *p* = 0.001) were significantly more common in patients with lower albumin levels. In line, the rates of severely reduced LVEF (<35%) increased progressively with lower albumin levels (Q1: 28.0% vs. Q4: 7.3%; *p* = 0.001) (Table 1).

The prevalence of CAD increased with lower albumin levels, with three-vessel disease present in 36.1%, 32.3%, 27.1%, and 21.4% in Q1 through Q4, respectively (*p* = 0.001). Percutaneous coronary intervention (PCI) was performed more frequently in patients with lower albumin levels (Q1: 45.2% vs. Q4: 36.5%; *p* = 0.001). Regarding laboratory values, lower albumin groups showed higher levels of CRP (Q1: 92.6 vs. Q4: 8.9 mg/L; *p* = 0.001) and N-terminal pro-B-type natriuretic peptide (NT-proBNP) (Q1: 4896 vs. 488 pg/mL; *p* = 0.001), alongside lower hemoglobin (Q1: 11.0 vs. Q4: 14.4 g/dL; *p* = 0.001) and eGFR (Q1: 58.5 vs. Q4: 74.3 mL/min/1.73 m^2^; *p* = 0.001). Finally, in-hospital all-cause mortality at 30 days increased markedly with lower albumin levels (Q1: 21.2% vs. Q4: 0.6%; *p* = 0.001).

### 3.2. Prognostic Impact of Albumin Levels in Patients Undergoing Coronary Angiography

**Figure 2:**
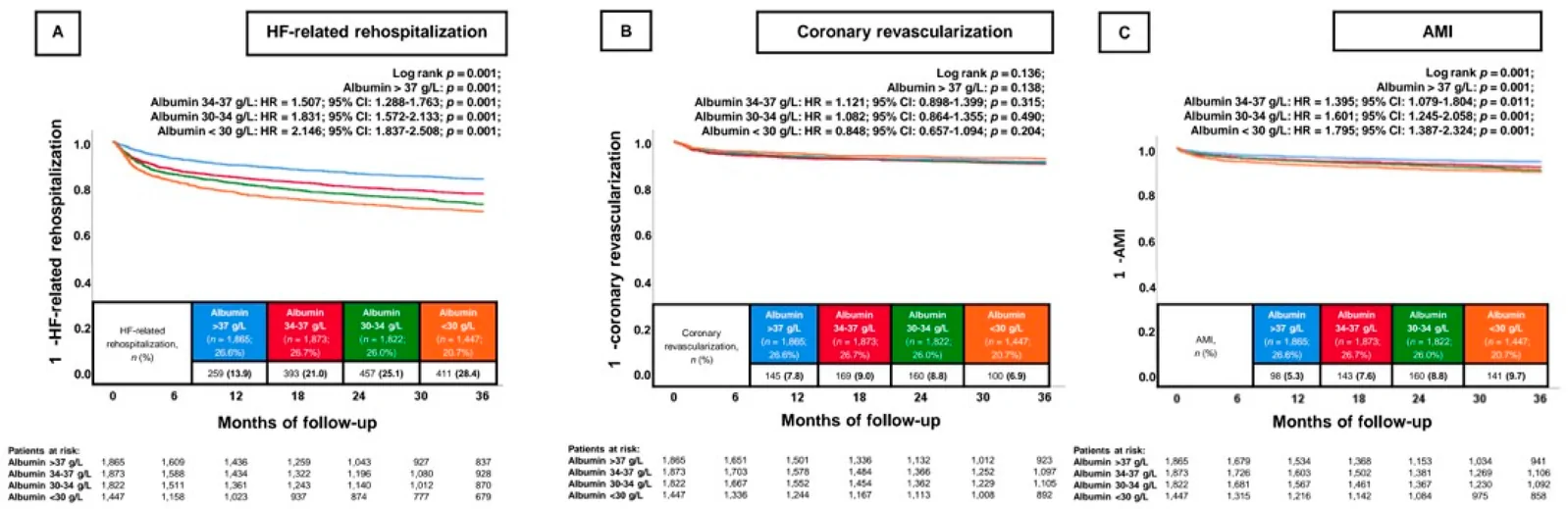
Kaplan–Meier curves illustrating the prognostic impact of albumin quartiles (Q1–Q4; Q4 as reference) in unselected patients undergoing coronary angiography on the risk of HF-related rehospitalization at 36 months (**A**), coronary revascularization at 36 months (**B**), and AMI at 36 months (**C**). Hazard ratios are reported relative to Q4 (albumin > 37 g/L). AMI, acute myocardial infarction; CI, confidence interval; HF, heart failure; HR, hazard ratio, Q, quartile.

The primary endpoint of HF-related rehospitalization at 36 months occurred in 28.4%, 25.1%, 21.0%, and 13.9% of patients in Q1 through Q4, respectively (*p* = 0.001). Coronary revascularization rates at 36 months did not differ significantly across albumin quartiles (Q1: 6.9%, Q2: 8.8%, Q3: 9.0%, Q4: 7.8%; *p* = 0.136). AMI at 36 months occurred in 9.7%, 8.8%, 7.6%, and 5.3% in Q1 through Q4, respectively (*p* = 0.001) (Table 2). Kaplan–Meier analyses revealed a significantly higher risk of HF-related rehospitalization at 36 months in patients with lower albumin levels compared to Q4 (Q3: HR = 1.507; 95% CI: 1.288–1.763; *p* = 0.001; Q2: HR = 1.831; 95% CI: 1.572–2.133; *p* = 0.001; Q1: HR = 2.146; 95% CI: 1.837–2.508; *p* = 0.001; log-rank *p* = 0.001) (Figure 2A). In contrast, no significant differences in the risk of coronary revascularization at 36 months were observed across albumin quartiles (log-rank *p* = 0.136) (Figure 2B). Lower albumin levels were, however, associated with a higher risk of AMI at 36 months (Q3: HR = 1.395; 95% CI: 1.079–1.804; *p* = 0.011; Q2: HR = 1.601; 95% CI: 1.245–2.058; *p* = 0.001; Q1: HR = 1.795; 95% CI: 1.387–2.324; *p* = 0.001; log-rank *p* = 0.001) (Figure 2C).

### 3.3. Multivariable Cox Regression Analyses

After multivariable adjustment, lower albumin levels were independently associated with a higher risk of HF-related rehospitalization at 36 months. Compared to Q4 (albumin > 37 g/L), patients in Q3 (albumin 34–37 g/L; HR = 1.319; 95% CI: 1.028–1.691; *p* = 0.029), Q2 (albumin 30–34 g/L; HR = 1.298; 95% CI: 1.038–1.623; *p* = 0.022), and Q1 (albumin < 30 g/L; HR = 1.381; 95% CI: 1.105–1.727; *p* = 0.005) were all at significantly higher risk (overall *p* = 0.043). In contrast, albumin levels were not independently associated with the risk of coronary revascularization (overall *p* = 0.927) or AMI (overall *p* = 0.252) at 36 months after multivariable adjustment.

Additional independent predictors of HF-related rehospitalization at 36 months included age (HR = 1.011; 95% CI: 1.006–1.017; *p* = 0.001), male sex (HR = 1.148; 95% CI 1.009–1.306; *p* = 0.036), diabetes mellitus (HR = 1.318; 95% CI: 1.165–1.493; *p* = 0.001), CKD (HR = 1.881; 95% CI: 1.562–2.265; *p* = 0.001), malignancy (HR = 1.290; 95% CI 1.035–1.607; *p* = 0.024), ADHF at index hospitalization (HR = 1.584; 95% CI: 1.375–1.824; *p* = 0.001), AF (HR = 1.245; 95% CI: 1.092–1.419; *p* = 0.001), and LVEF < 35% (HR = 2.505; 95% CI: 2.179–2.879; *p* = 0.001) (Table 3).

### 3.4. Prognostic Impact of Albumin Levels in Pre-Specified Subgroups

The association between lower albumin levels and higher risk of HF-related rehospitalization at 36 months was particularly evident in patients ≥70 years of age (*p* = 0.041), patients with LVEF ≥ 35% (*p* = 0.049), and patients with 0- to 1-vessel CAD (*p* = 0.045). In contrast, no significant association was observed within the remaining individual strata (all *p* > 0.07). Formal interaction terms (albumin x subgroup) were significant for age, LVEF category, indication acuity, and ADHF status (all *p* < 0.005), and remained significant after Bonferroni correction (α = 0.0071); interactions for sex, multivessel CAD extent, and STEMI status were not significant (Table 4). In the stratified analysis by indication acuity, the adjusted HR for albumin was 0.692 (95% CI 0.342–1.399; *p* = 0.305) in the acute subgroup and 0.607 (95% CI 0.317–1.163; *p* = 0.132) in the elective subgroup.

### 3.5. Discriminative Performance

Discrimination for HF-related rehospitalization was evaluated using the AHEAD score, both alone and in combination with albumin. The AHEAD score alone yielded an AUC of 0.650 (95% CI 0.635–0.665), while the addition of albumin increased the AUC only marginally, to 0.655 (95% CI 0.640–0.670). Albumin alone yielded a lower discriminative value, with an AUC of 0.596 (95% CI 0.580–0.612), consistent with its role as an independent prognostic marker rather than a standalone risk-stratification tool.

## 4. Discussion

The main findings investigating the association of albumin levels among consecutive patients undergoing CA can be summarized as follows. Lower albumin levels were associated with a greater burden of comorbidities and a greater extent of CAD and independently predicted the risk of HF-related rehospitalization at 36 months. In pre-specified subgroup analyses using albumin as a continuous variable, this association was most robust in patients ≥70 years of age and in those with LVEF ≥ 35%; however these subgroup findings should be interpreted as hypothesis-generating given the number of comparisons performed.

Albumin is the most abundant circulating plasma protein, contributing to colloid osmotic pressure, transport of endogenous and exogenous compounds, antioxidant defense, and the modulation of inflammatory pathways [1,2]. Hypoalbuminemia in cardiovascular patients may rarely reflect a single underlying cause but rather the convergence of systemic inflammation, malnutrition, hepatic congestion, and renal losses, all common in advanced CAD or HF [3,16]. The inverse relationship between albumin and inflammatory markers observed here, including markedly elevated CRP and NT-proBNP in lower albumin groups, is consistent with its role as a negative acute-phase protein whose hepatic synthesis is suppressed during inflammatory states [1,17,18,19], reflecting the close interplay between hypoalbuminemia, neurohormonal activation, and hemodynamic congestion in this unselected CA cohort. Congestion-related electrolyte disturbances, particularly involving sodium and chloride handling, have similarly been associated with adverse outcomes in acute HF and may represent a complementary prognostic pathway, especially in patients with renal dysfunction [20,21]. The present study showed a clear gradient in CAD prevalence and severity across albumin groups, with three-vessel disease present in 36.1%, 32.3%, 27.1%, and 21.4% in Q1 through Q4, respectively, corroborating prior reports of an inverse relationship between albumin and angiographic CAD severity. Narang et al. reported a comparable association in patients undergoing elective CA [22], Deveci and Gazi demonstrated that albumin independently predicted both the presence and complexity of CAD [23], and Joki et al. showed that pre-dialysis albumin levels were inversely related to coronary atherosclerosis severity in end-stage renal disease [14]. Furthermore, Kurtul et al. demonstrated that lower albumin independently predicted a higher SYNTAX score in ACS patients undergoing CA, providing a direct mechanistic link between systemic hypoalbuminemia and the extent of coronary atherosclerosis [24]. These associations likely reflect albumin’s antioxidant and anti-inflammatory properties, which may attenuate endothelial dysfunction and slow atherosclerotic plaque progression [1,3]. The higher rates of PCI in lower albumin groups further support hypoalbuminemia as a marker of more advanced, diffuse coronary disease requiring more extensive revascularization, consistent with prior analyses from the present registry examining other prognostic markers in this same unselected CA population, including diabetes mellitus, age, obesity, anemia, CKD, LVEF, and AF subtypes [25,26,27,28,29,30,31].

The independent association between lower albumin and HF-related rehospitalization observed here extends prior evidence from selected CAD cohorts. Suzuki et al. showed that lower albumin independently predicted long-term cardiovascular death and HF hospitalization in stable CAD patients undergoing PCI [7], while Wada et al. reported in two related analyses that albumin independently predicted long-term outcomes in a broader PCI population and specifically among those with preserved renal function [8,32]. More recently, Yazdani et al. demonstrated an independent association between carbamylated albumin and HF and mortality in patients undergoing CA [33]. The present study extends these observations to a larger, consecutive all-comer cohort spanning the full spectrum of CA indications, providing a more generalizable estimate of albumin’s prognostic value in contemporary practice. The apparent monotonic gradient between albumin levels and HF-related rehospitalization observed in the present study is broadly consistent with prior dose–response evidence. However, formal assessment of nonlinearity using restricted cubic splines was not performed, and a recent large analysis suggested that the prognostic benefit of albumin normalization may plateau above the normal reference threshold [34]; future analyses should explore potential threshold effects. As a linear approximation of this relationship, log-transformed albumin was additionally modeled as a continuous covariate in the fully adjusted model; each unit increase in ln-albumin was associated with an adjusted HR = 0.619 (95% CI 0.385–0.994; *p* = 0.047) for HF-related rehospitalization, consistent in direction with the quartile-based analysis, though this linear model does not formally test for nonlinearity. The numerical similarity of the adjusted HRs across Q1 to Q3 relative to Q4 may reflect a threshold-type relationship rather than a strictly linear gradient.

Notably, after multivariable adjustment, albumin was not independently associated with coronary revascularization or AMI, suggesting that its prognostic impact is channeled specifically through HF-related pathways rather than recurrent ischemic mechanisms. This is biologically plausible: albumin reflects systemic inflammation, malnutrition, and neurohormonal activation, established drivers of myocardial dysfunction and HF progression, rather than plaque instability or thrombotic risk per se [1,13,16]. Although this pattern contrasts with findings from the HFmrEF registry of our group, where albumin independently predicted long-term all-cause mortality but not HF-related rehospitalization [11], this discrepancy may reflect the competing risk of mortality in an already-established HFmrEF population, where death may preempt rehospitalization as the dominant downstream event. The association between hypoalbuminemia and adverse HF outcomes is well-established across phenotypes. Horwich et al. showed that albumin independently predicted survival in systolic HF [9], a comparable association reported by Armentaro et al. in a broader chronic HF population [35], and a systematic review and meta-analysis by El Iskandarani et al. confirmed significantly higher short- and long-term mortality with hypoalbuminemia across diverse HF populations [10]. Most directly relevant to the present endpoint, Yoshioka et al. showed that lower admission albumin independently predicted incident HF following AMI [36]. More recently, Biancucci et al. confirmed this association in a comprehensive review, with supporting evidence from the TOPCAT trial in HFpEF patients [37,38], while Ghazal and Khalife similarly emphasized hypoalbuminemia as an integrative marker of nutritional status, systemic inflammation, and end-organ dysfunction, warranting its routine incorporation into HF risk stratification [13].

The finding that albumin’s prognostic impact on HF-related rehospitalization was specifically evident in patients with LVEF ≥ 35% but not LVEF < 35% deserves further attention. Patients with severely reduced LVEF represent a high-risk phenotype in whom competing risks, including sudden cardiac death and progressive pump failure, may attenuate albumin’s relative contribution as a prognostic discriminator. In contrast, patients with mildly reduced or preserved ejection fraction, in whom HF is driven more by diastolic dysfunction, inflammation, and neurohormonal activation, may be more susceptible to the adverse pathophysiological effects of hypoalbuminemia. Furthermore, the substantially higher in-hospital mortality observed in patients with severely reduced LVEF, reaching 21.2% in Q1 compared to 0.6% in Q4, likely introduces a relevant competing risk, whereby early death during the index hospitalization may preclude subsequent HF-related rehospitalization and thereby attenuate the observable prognostic signal of albumin in this subgroup. This may at least partly explain the absence of a significant albumin-outcome association in patients with LVEF < 35% (overall *p* = 0.566) and underscores the need for formal competing risk analyses in future studies.

Formal interaction terms provided additional context for these subgroup findings, and we discuss them in relation to the interaction tests rather than individual-stratum *p*-values considered in isolation, consistent with established caution against overinterpreting subgroup findings without regard to the interaction itself [39]. Two subgroups showed both a significant interaction and a significant association within at least one corresponding stratum: age and LVEF category. The association between albumin and HF-related rehospitalization was significant in patients ≥70 years of age but not in younger patients, consistent with evidence that hypoalbuminemia carries particular prognostic weight in older patients, in whom it more directly reflects an accumulation of frailty, malnutrition, and chronic inflammatory burden [40]. The association was similarly significant in patients with LVEF ≥ 35% but not in those with LVEF < 35%, consistent with growing recognition that albumin carries distinct pathophysiological significance across the ejection fraction spectrum: in HFpEF and related preserved-function phenotypes, hypoalbuminemia has been linked to myocardial fibrosis and adverse pulsatile hemodynamics and remains independently prognostic even after adjustment for natriuretic peptides [41], whereas in patients with LVEF < 35%, its prognostic signal may be attenuated by the competing risk of early mortality discussed above. Although albumin was significantly associated with the outcome in the 0- to 1-vessel CAD stratum alone, the corresponding interaction was not significant (*p* = 0.726), an isolated finding that should not be interpreted as a true difference by CAD extent [39]. Despite significant interactions for indication acuity and ADHF status, albumin was not significantly associated with the outcome within either individual stratum for these variables, more consistent with reduced power following stratification than a true absence of association [42]; no significant interaction was observed for sex or STEMI status.

From a clinical perspective, routine albumin measurement at the time of CA may provide incremental prognostic information beyond standard clinical and angiographic variables, particularly for identifying patients at elevated risk of HF-related rehospitalization. Given its universal availability, negligible cost, and routine measurement in most hospitalized patients, integrating albumin into post-CA risk stratification appears both feasible and clinically meaningful.

Taken together, the sensitivity analyses summarized above reinforce the primary finding that albumin is independently and robustly associated with HF-related rehospitalization,. The observation that this robust association did not translate into improved discrimination when added to the AHEAD score is consistent with the well-recognized methodological principle that the AUC is a relatively insensitive metric for detecting the incremental value of a single new marker, particularly one correlated with established risk factors already in the model [43]. This distinction between statistical association and clinical discrimination indicates that albumin may be regarded as a robust prognostic marker rather than a standalone tool for individual risk stratification in this population.

## 5. Study Limitations

The present study has several limitations. Firstly, all data, including follow-up data, were sourced solely from hospital records of a single institution. Because patients were identified using OPS codes, there may be lower documented event rates of pre-existing comorbidities, and events occurring at other institutions during follow-up were not captured. Secondly, a major limitation was the unavailability of long-term all-cause mortality data beyond index hospitalization. Given the substantially higher in-hospital mortality in the lowest albumin group, formal competing risk analyses using Fine–Gray models should be addressed in future studies. We considered, as an alternative to formal competing-risk modeling, excluding patients who died within a fixed early window, but decided against this: because early mortality is concentrated in the lowest albumin quartile, such an exclusion would selectively remove the most severely ill patients from precisely the group of primary interest, exchanging an unaddressed competing risk for a survivorship bias in the opposite direction. Because standard Cox models treat death as non-informative censoring, this differential early mortality may distort the estimated association for HF-related rehospitalization in this subgroup in a direction that cannot be predicted from theoretical considerations alone. For this reason, we considered a Fine–Gray subdistribution hazards model, which treats death as a competing event rather than assuming it away, methodologically preferable to an ad hoc exclusion rule for this cohort; this remains an acknowledged limitation of the present cause-specific analysis. Thirdly, serum albumin was measured at a single time point during index hospitalization, and serial measurements as well as data on post-discharge treatment changes were not available. Furthermore, due to the retrospective and single-center study design, the results may have been influenced by measured and unmeasured confounding factors, despite multivariable Cox regression analyses, other unmeasured factors not captured as continuous covariates. All models were adjusted for CRP and malignancy; albumin remained independently associated with HF-related rehospitalization even after accounting for these factors, with malignancy itself also independently associated with the outcome, while CRP was not, indicating that albumin’s prognostic association is not simply a proxy for the systemic inflammation or malignancy burden captured by these two markers. As with any observational analysis, residual confounding by other unmeasured factors cannot be entirely excluded, and may not be generalizable to broader populations outside of Germany.

## 6. Conclusions

In unselected patients undergoing CA, lower albumin independently predicted HF-related rehospitalization at 36 months, with no independent association observed for coronary revascularization or AMI. The prognostic signal was most pronounced in patients ≥70 years of age and in those with LVEF ≥ 35%, findings that should be interpreted as hypothesis-generating given the number of subgroup comparisons performed. These findings suggest that albumin’s prognostic impact operates through the inflammatory and neurohormonal rather than ischemic pathways, and that its effect is most discriminating in hemodynamically stable patients with preserved or mildly reduced systolic function. Given its universal availability, negligible cost, and routine measurement in hospitalized patients, albumin represents a readily implementable tool that was independently and robustly associated with HF-related rehospitalization risk across multiple sensitivity analyses, though it did not improve discrimination beyond the AHEAD score in the present cohort. Future prospective studies should evaluate whether the targeted correction of hypoalbuminemia can modify the long-term risk of HF-related rehospitalization in this population.

## Figures and Tables

**Table 1:** Baseline characteristics.

	Q1 (*n* = 1872) Albumin < 30 g/L		Q2 (*n* = 1882) Albumin 30–34 g/L		Q3 (*n* = 1890 Albumin 34–37 g/L		Q4 (*n* = 1876) Albumin > 37 g/L		*p* Value
Age, median (IQR)	73	(64–81)	72	(62–80)	69	(58–78)	64	(55–73)	**0.001**
Male sex, *n* (%)	1164	(62.2)	1178	(62.6)	1234	(65.3)	1314	(70.0)	**0.001**
Body mass index, kg/m^2^, median (IQR)	26	(23–30)	27	(24–31)	28	(25–31)	28	(25–31)	**0.001**
**Cardiovascular risk factors**, *n* (%)									
Arterial hypertension	1396	(74.6)	1704	(90.5)	1697	(89.8)	1618	(86.2)	**0.001**
Diabetes mellitus	663	(35.4)	626	(33.3)	541	(28.6)	441	(23.5)	**0.001**
Hyperlipidemia	445	(23.8)	648	(34.4)	765	(40.5)	841	(44.8)	**0.001**
**Prior medical history**, *n* (%)									
Coronary artery disease	251	(13.4)	242	(12.9)	250	(13.2)	238	(12.7)	0.909
Myocardial infarction	42	(2.2)	32	(1.7)	27	(1.4)	23	(1.2)	0.080
PCI	32	(1.7)	69	(3.7)	74	(3.9)	91	(4.9)	**0.001**
CABG	170	(9.1)	135	(7.2)	111	(5.9)	111	(5.9)	**0.001**
Congestive HF	213	(11.4)	163	(8.7)	157	(8.3)	129	(6.9)	**0.001**
Pacemaker	25	(1.3)	27	(1.4)	30	(1.6)	32	(1.7)	0.799
COPD	115	(6.1)	74	(3.9)	64	(3.4)	40	(2.1)	**0.001**
Chronic kidney disease	185	(9.9)	101	(5.4)	87	(4.6)	40	(2.1)	**0.001**
Liver cirrhosis	40	(2.1)	20	(1.1)	11	(0.6)	9	(0.5)	**0.001**
Malignancy	180	(9.6)	122	(6.5)	73	(3.9)	60	(3.2)	**0.001**
Stroke	20	(1.1)	17	(0.9)	8	(0.4)	14	(0.7)	0.140
**Comorbidities at index hospitalization**, *n* (%)									
ACS	912	(48.7)	1104	(58.7)	1123	(59.4)	1141	(60.8)	
Unstable angina	189	(10.1)	431	(22.9)	584	(30.9)	793	(42.3)	**0.001**
STEMI	284	(15.2)	285	(15.1)	210	(11.1)	126	(6.7)	**0.001**
NSTEMI	439	(23.5)	388	(20.6)	329	(17.4)	222	(11.8)	**0.001**
Atrial fibrillation	576	(30.8)	521	(27.7)	476	(25.2)	410	(21.9)	**0.001**
Paroxysmal AF	166	(9.6)	148	(8.3)	145	(7.9)	151	(8.3)	**0.006**
Persistent AF	62	(3.6)	57	(3.2)	68	(3.7)	50	(2.7)	
Permanent AF	63	(3.6)	80	(4.5)	76	(4.1)	49	(2.7)	
New-onset AF	143	(8.3)	129	(7.3)	131	(7.1)	104	(5.7)	
Atrial flutter	34	(1.8)	45	(2.4)	44	(2.3)	35	(1.9)	0.478
ADHF	365	(19.5)	276	(14.7)	187	(9.9)	94	(5.0)	**0.001**
Cardiogenic shock	251	(13.4)	46	(2.4)	12	(0.6)	11	(0.6)	**0.001**
Atrioventricular block	65	(3.5)	58	(3.1)	41	(2.2)	28	(1.5)	**0.001**
Cardiopulmonary resuscitation	411	(22.0)	89	(4.7)	28	(1.5)	17	(0.9)	**0.001**
Out-of-hospital	294	(15.7)	60	(3.2)	19	(1.0)	6	(0.3)	**0.001**
In-hospital	117	(6.3)	29	(1.5)	9	(0.5)	11	(0.6)	**0.001**
Valvular heart disease	353	(18.9)	362	(19.2)	304	(16.1)	254	(13.5)	**0.001**
Stroke	108	(5.8)	77	(4.1)	65	(3.4)	36	(1.9)	**0.001**
**LVEF**, *n* (%)									
>55	496	(32.4)	776	(44.7)	939	(53.1)	1054	(61.2)	**0.001**
54–45	310	(20.2)	424	(24.4)	418	(23.7)	364	(21.1)	
44–35	297	(19.4)	266	(15.3)	212	(12.0)	179	(10.4)	
<35	429	(28.0)	271	(15.6)	198	(11.2)	126	(7.3)	
Not documented	340	(18.2)	145	(7.7)	123	(6.5)	153	(8.2)	

ACS, acute coronary syndrome; ADHF, acute decompensated heart failure; AF, atrial fibrillation; CABG, coronary artery bypass grafting; COPD, chronic obstructive pulmonary disease;. IQR, interquartile range; LVEF, left ventricular ejection fraction; NSTEMI, non-ST-segment elevation myocardial infarction; PCI, percutaneous coronary intervention; Q, quartile; STEMI, ST-segment elevation myocardial infarction. Albumin quartile cut-offs: Q1 < 30 g/L; Q2 30–34 g/L; Q3 34–37 g/L; Q4 > 37 g/L. Values are expressed as median (IQR) or number (percentage). Level of significance: *p* ≤ 0.05. Bold type indicates statistical significance.

**Table 2:** Procedural, laboratory, and follow-up data.

	Q1 (*n* = 1872) Albumin < 30 g/L		Q2 (*n* = 1882) Albumin 30–34 g/L		Q3 (*n* = 1890) Albumin 34–37 g/L		Q4 (*n* = 1876) Albumin > 37 g/L		*p* Value
**Coronary angiography**, *n* (%)									
No evidence of coronary artery disease	465	(24.8)	489	(26.0)	602	(31.9)	729	(38.9)	**0.001**
1-vessel disease	324	(17.3)	386	(20.5)	395	(20.9)	397	(21.2)	
2-vessel disease	408	(21.8)	399	(21.2)	380	(20.1)	348	(18.6)	
3-vessel disease	675	(36.1)	608	(32.3)	513	(27.1)	402	(21.4)	
Right coronary artery	1014	(54.2)	956	(50.8)	832	(44.0)	695	(37.0)	**0.001**
Left main trunk	247	(13.2)	230	(12.2)	192	(10.2)	161	(8.6)	**0.001**
Left anterior descending	1126	(60.1)	1089	(57.9)	983	(52.0)	891	(47.5)	**0.001**
Left circumflex	919	(49.1)	836	(44.4)	759	(40.2)	619	(33.0)	**0.001**
Ramus intermedius	262	(14.0)	227	(12.1)	209	(11.1)	139	(7.4)	**0.001**
CABG	73	(3.9)	73	(3.9)	50	(2.6)	27	(1.4)	**0.001**
Chronic total occlusion	189	(10.1)	164	(8.7)	125	(6.6)	121	(6.4)	**0.001**
**PCI**, *n* (%)	846	(45.2)	893	(47.4)	807	(42.7)	685	(36.5)	**0.001**
Right coronary artery	341	(18.2)	334	(17.7)	327	(17.3)	256	(13.6)	**0.001**
Left main trunk	86	(4.6)	81	(4.3)	67	(3.5)	52	(2.8)	**0.016**
Left anterior descending	456	(24.4)	456	(24.2)	413	(21.9)	360	(19.2)	**0.001**
Left circumflex	300	(16.0)	305	(16.2)	258	(13.7)	206	(11.0)	**0.001**
Ramus intermedius	34	(1.8)	39	(2.1)	35	(1.9)	27	(1.4)	0.531
CABG	16	(0.9)	25	(1.3)	9	(0.5)	5	(0.3)	**0.001**
**Sent to CABG**, *n* (%)	78	(4.2)	92	(4.9)	71	(3.8)	90	(4.8)	0.276
**Procedural data**									
Number of stents, median (IQR)	2	(1–4)	2	(1–3)	2	(1–3)	2	(1–3)	**0.001**
Stent length, mm, median (IQR)	50	(28–85)	43	(24–76)	42	(24–72)	38	(23–68)	**0.001**
Contrast, ml, median (IQR)	128	(75–218)	126	(75–201)	110	(70–191)	98	(60–165)	**0.001**
**Baseline laboratory values**, median (IQR)									
Sodium, mmol/L	140	(137–142)	139	(138–141)	139	(138–141)	139	(138–141)	**0.001**
Potassium, mmol/L	4.0	(3.8–4.3)	3.9	(3.7–4.2)	3.9	(3.7–4.2)	3.9	(3.7–4.2)	**0.001**
Calcium, mmol/L	2.1	(2.0–2.2)	2.2	(2.1–2.3)	2.2	(2.2–2.3)	2.3	(2.2–2.4)	**0.001**
Creatinine, mg/dL	1.3	(0.9–1.9)	1.0	(0.8–1.3)	1.0	(0.8–1.2)	1.0	(0.8–1.1)	**0.001**
eGFR, mL/min/1.73 m^2^	58.5	(37–83)	66.5	(49–84)	70.6	(55–84)	74.3	(61–86)	**0.001**
Urea, mg/dL	53.1	(37–80)	39.1	(30–53)	35.5	(29–46)	33.1	(27–41)	**0.001**
Hemoglobin, g/dL	11.0	(10–12)	12.9	(12–14)	13.7	(13–15)	14.4	(13–15)	**0.001**
WBC count, x 10^9^/L	11.2	(8.4–14.5)	9.1	(7.3–11.2)	8.5	(6.9–10.3)	8.0	(6.6–9.7)	**0.001**
Platelet count, x 10^9^/L	250	(193–316)	234	(192–285)	230	(191–272)	231	(193–271)	**0.001**
HbA1c, %	5.9	(5.5–7.0)	5.9	(5.5–6.7)	5.8	(5.5–6.5)	5.7	(5.4–6.3)	**0.001**
LDL-cholesterol, mg/dL	86	(64–112)	103	(78–132)	112	(86–140)	118	(87–149)	**0.001**
HDL-cholesterol, mg/dL	38.5	(30–49)	43.0	(35–53)	42.0	(35–52)	44.0	(37–55)	**0.001**
Triglycerides, mg/dL	126	(94–174)	119	(90–165)	129	(96–178)	133	(96–194)	**0.001**
C-reactive protein, mg/L	92.6	(41–165)	23.5	(9.2–60)	12.6	(6.2–28)	8.9	(5.3–17)	**0.001**
Procalcitonin, µg/L	0.7	(0.2–2.9)	0.1	(0.1–0.4)	0.1	(0.0–0.2)	0.1	(0.0–0.1)	**0.001**
INR	1.1	(1.0–1.3)	1.1	(1.0–1.1)	1.0	(1.0–1.1)	1.0	(1.0–1.1)	**0.001**
NT-pro BNP, pg/mL	4896	(1943–12,960)	2192	(672–5287)	1129	(295–3016)	488	(127–2085)	**0.001**
Cardiac troponin I, µg/L	1.3	(0.2–7.6)	0.7	(0.1–6.0)	0.4	(0.1–3.7)	0.2	(0.1–2.9)	**0.001**
CK, U/L	209	(48–604)	131	(79–315)	124	(78–223)	123	(86–202)	**0.001**
CK-MB, U/L	45.0	(27–92)	35.0	(22–73)	28.8	(20–54)	24.0	(18–37)	**0.001**
**Medication at discharge**, *n* (%)									
ACE-inhibitor	721	(49.8)	1005	(55.2)	996	(53.2)	858	(46.0)	**0.001**
ARB	267	(18.5)	462	(25.4)	449	(24.0)	497	(26.6)	**0.001**
Beta-blocker	1058	(73.1)	1377	(75.6)	1340	(71.5)	1202	(64.5)	**0.001**
MRA	271	(18.7)	311	(17.1)	251	(13.4)	226	(12.1)	**0.001**
ARNI	21	(1.5)	17	(0.9)	23	(1.2)	16	(0.9)	0.334
SGLT2-inhibitor	46	(3.2)	86	(4.7)	99	(5.3)	113	(6.1)	**0.002**
Statin	1014	(70.1)	1397	(76.7)	1414	(75.5)	1364	(73.1)	**0.001**
ASA	926	(64.0)	1218	(66.8)	1218	(65.0)	1170	(62.7)	0.065
P2Y12-inhibitor	700	(48.4)	966	(53.0)	895	(47.8)	766	(41.1)	**0.001**
DOAC	417	(28.8)	559	(30.7)	541	(28.9)	446	(23.9)	**0.001**
**Follow-up data**, median (IQR)									
Hospitalization time	14.0	(7.0–23)	8.0	(5.0–13)	6.0	(4.0–9.0)	4.0	(3.0–7.0)	**0.001**
ICU time	0.0	(0.0–1.0)	0.0	(0.0–0.0)	0.0	(0.0–0.0)	0.0	(0.0–0.0)	**0.001**
**All-cause mortality, 30 days**, *n* (%)	397	(21.2)	60	(3.2)	16	(0.8)	11	(0.6)	**0.001**
**Primary endpoint**, *n* (%)									
HF-related rehospitalization, at 36 months	411	(28.4)	457	(25.1)	393	(21.0)	259	(13.9)	**0.001**
**Secondary endpoints**, *n* (%)									
Coronary revascularization, at 36 months	100	(6.9)	160	(8.8)	169	(9.0)	145	(7.8)	0.136
Acute myocardial infarction, at 36 months	141	(9.7)	160	(8.8)	143	(7.6)	98	(5.3)	**0.001**

ACE, angiotensin-converting enzyme; ARB, angiotensin receptor blocker; ARNI, angiotensin receptor neprilysin inhibitor; ASA, acetylsalicylic acid; CABG, coronary artery bypass grafting; CK, creatine kinase; CK-MB, creatine kinase myocardial band; DOAC, direct oral anticoagulant; eGFR, estimated glomerular filtration rate; HbA1c, glycated hemoglobin; HDL, high-density lipoprotein; HF, heart failure; ICU, intensive care unit; INR, international normalized ratio; IQR, interquartile range; LDL, low-density lipoprotein; MRA, mineralocorticoid receptor antagonists; NT-pro BNP, N-terminal pro-B-type natriuretic peptide; PCI, percutaneous coronary intervention; Q, quartile; SGLT2, sodium–glucose cotransporter 2; WBC, white blood cell count. Albumin quartile cut-offs: Q1 < 30 g/L; Q2 30–34 g/L; Q3 34–37 g/L; Q4 > 37 g. Level of significance: *p* ≤ 0.05. Bold type indicates statistical significance. For the primary and secondary endpoints, percentages were calculated among patients with available follow-up data after exclusion of those who died during index hospitalization (follow-up *n*: Albumin > 37 g/L, *n* = 1865; Albumin 34–37 g/L, *n* = 1873; Albumin 30–34 g/L, *n* = 1822; Albumin < 30 g/L, *n* = 1447).

**Table 3:** Multivariate Cox regression analyses with regard to risk of heart-failure-related rehospitalization, coronary revascularization, and acute myocardial infarction at 36 months.

	HF-Related Rehospitalization			Coronary Revascularization			AMI		
Variables	HR	95% CI	*p* Value	HR	95% CI	*p* Value	HR	95% CI	*p* Value
Age	1.011	1.006–1.017	**0.001**	0.998	0.989–1.007	0.706	0.989	0.981–0.998	**0.015**
Male sex	1.148	1.009–1.306	**0.036**	1.536	1.211–1.947	**0.001**	1.169	0.934–1.462	0.173
Body mass index	1.005	0.994–1.015	0.410	1.005	0.986–1.024	0.621	0.998	0.980–1.016	0.810
Diabetes mellitus	1.318	1.165–1.493	**0.001**	1.441	1.160–1.790	**0.001**	1.338	1.084–1.652	**0.007**
Chronic kidney disease	1.881	1.562–2.265	**0.001**	1.310	0.878–1.953	0.186	1.058	0.729–1.537	0.766
Malignancy	1.290	1.035–1.607	**0.024**	1.166	0.769–1.767	0.471	1.383	0.939–2.037	0.101
Anemia	1.144	0.996–1.314	0.056	0.997	0.778–1.276	0.978	1.044	0.824–1.323	0.719
CRP	1.005	0.948–1.065	0.877	0.952	0.861–1.053	0.341	0.880	0.796–0.973	**0.013**
STEMI	1.026	0.836–1.258	0.809	2.012	1.510–2.682	**0.001**	1.160	0.833–1.616	0.378
NSTEMI	0.959	0.825–1.116	0.589	1.882	1.479–2.394	**0.001**	1.584	1.249–2.007	**0.001**
Acute decompensated heart failure	1.584	1.375–1.824	**0.001**	0.908	0.662–1.246	0.551	1.481	1.160–1.892	**0.002**
Atrial fibrillation	1.245	1.092–1.419	**0.001**	1.051	0.818–1.351	0.697	1.140	0.905–1.436	0.265
LVEF < 35%	2.505	2.179–2.879	**0.001**	0.859	0.628–1.175	0.342	3.394	2.717–4.239	**0.001**
Albumin > 37 g/L (Reference group)									
Albumin 34–37 g/L	1.319	1.028–1.691	**0.029**	0.965	0.646–1.442	0.863	1.402	0.937–2.096	0.100
Albumin 30–34 g/L	1.298	1.038–1.623	**0.022**	1.048	0.749–1.466	0.785	1.163	0.813–1.664	0.409
Albumin < 30 g/L	1.381	1.105–1.727	**0.005**	1.063	0.764–1.480	0.717	1.328	0.936–1.883	0.112

AMI, acute myocardial infarction; CI, confidence interval; CRP, C-reactive protein; HF, heart failure; HR, hazard ratio; LVEF, left ventricular ejection fraction; NSTEMI, non-ST-elevation myocardial infarction; STEMI, ST-elevation myocardial infarction. Level of significance: *p* ≤ 0.05. Bold type indicates statistical significance.

**Table 4:** Multivariable Cox regression by subgroup with albumin modeled as a continuous (ln-transformed) variable, and formal interaction tests.

Subgroup	*n* (Events, %)	Adjusted HR	95% CI	*p* Value	Interaction *p* Value
Age					
<70 years	3776 (661, 17.5)	0.832	0.445–1.555	0.565	**0.001**
≥70 years	3916 (444, 11.3)	0.452	0.211–0.969	**0.041**	
Sex					
Male	4993 (739, 14.8)	0.675	0.374–1.219	0.193	0.684
Female	2699 (366, 13.6)	0.479	0.213–1.074	0.074	
CAD extent					
0- to 1-vessel disease	3887 (447, 11.5)	0.480	0.235–0.982	**0.045**	0.726
Multivessel disease	3825 (658, 17.2)	0.701	0.369–1.333	0.279	
LVEF					
≥35%	5850 (778, 13.3)	0.571	0.327–0.997	**0.049**	**0.001**
<35%	1038 (327, 31.5)	0.915	0.360–2.331	0.853	
Indication acuity					
Elective	3706 (514, 16.7)	0.607	0.317–1.163	0.132	**0.001**
Acute *	4616 (591, 12.8)	0.692	0.342–1.399	0.305	
STEMI					
Absent	909 (119, 13.1)	0.730	0.130–4.095	0.720	0.873
Present	1382 (235, 17.0)	0.494	0.171–1.426	0.192	
ADHF					
Absent	927 (330, 35.6)	0.702	0.287–1.714	0.437	**0.005**
Present	6765 (775, 11.5)	0.648	0.368–1.139	0.132	

* Acute indications: unstable angina, NSTEMI, STEMI, resuscitated cardiac arrest, or cardiogenic shock. Adjusted HR is per unit increase in ln-albumin, from Cox proportional hazards models adjusted for age, sex, BMI, diabetes, CKD, malignancy, anemia, CRP, STEMI, NSTEMI, ADHF, AF, and LVEF < 35% (subgroup-defining variable excluded from its own stratified model). ADHF, acute decompensated heart failure; CAD, coronary artery disease; CKD, chronic kidney disease; CI, confidence interval; CRP, C-reactive protein; HR, hazard ratio; LVEF, left ventricular ejection fraction; NSTEMI, non-ST-elevation myocardial infarction; STEMI, ST-elevation myocardial infarction. Level of significance: *p* ≤ 0.05. Bold type indicates statistical significance.

## Data Availability

The data presented in this study are available on request from the corresponding author. The data are not publicly available due to data protection regulations and the retrospective single-center registry design.
